# Patients’ well-being during the transition period after psychiatric hospitalization to school: insights from an intensive longitudinal assessment of patient–parent–teacher triads

**DOI:** 10.1186/s40359-023-01197-0

**Published:** 2023-06-16

**Authors:** Marlene Finkbeiner, Lena-Marie Wahl, Jan Kühnhausen, Johanna Schmid, Leona Hellwig, Vera Brenner, Ute Dürrwächter, Annette Conzelmann, Augustin Kelava, Tobias J. Renner, Caterina Gawrilow

**Affiliations:** 1 Department of Child and Adolescent Psychiatry, Psychosomatics and Psychotherapy, University Hospital of Psychiatry and Psychotherapy, Tuebingen, Germany; 2 Department of Psychology, University of Tuebingen, TübingenTuebingen, Germany; 3 Methods Center, University of Tuebingen, Tuebingen, Germany; 4 LEAD Graduate School and Research Network, University of Tuebingen, Tuebingen, Germany; 5 Department of Psychology (Clinical Psychology II), PFH – Private University of Applied Sciences, Goettingen, Germany; 6 German Center for Mental Health (DZPG), Tuebingen, Germany

**Keywords:** Ambulatory assessment, Patient–parent–teacher triad, Psychiatric hospital to school transition, Reintegration, Well-being, Within-person

## Abstract

**Background:**

The transition period after psychiatric hospitalization back to school is accompanied by various challenges, including a substantial risk for rehospitalization. Self-efficacy and self-control, as transdiagnostic variables and important predictors of coping with school demands, should be crucial factors for successful adaptation processes as well as an overall high well-being during school reentry. The present study therefore investigates how patients’ well-being develops during this period, and how it is related to patients’ self-control and academic self-efficacy, as well as parents’ and teachers’ self-efficacy in dealing with the patient.

**Methods:**

In an intensive longitudinal design, daily ambulatory assessment measures via smartphone were collected with self-reports from the triadic perspective of 25 patients (*M*_*age*_ = 10.58 years), 24 parents, and 20 teachers on 50 consecutive school days, starting 2 weeks before discharge from a psychiatric day hospital (mean compliance rate: 71% for patients, 72% for parents and 43% for teachers). Patients answered daily questions between five and nine o'clock in the evening about their well-being, self-control, academic self-efficacy and about positive and negative events at school, as well as parents and teachers about their self-efficacy in dealing with the patient.

**Results:**

Multilevel modeling revealed that on average, patients’ well-being and self-control decreased during the transition period, with trends over time differing significantly between patients. While patients’ academic self-efficacy did not systematically decrease over time, it did show considerable intra-individual fluctuation. Importantly, patients experienced higher well-being on days with higher self-control and academic self-efficacy as well as with higher parental self-efficacy. Daily teacher self-efficacy did not show a significant within-person relationship to daily patients’ well-being.

**Conclusions:**

Well-being in the transition period is related to self-control and self-efficacy of patients and their parents. Thus, addressing patients’ self-control and academic self-efficacy, as well as parental self-efficacy, seems promising to enhance and stabilize well-being of patients during transition after psychiatric hospitalization.

*Trial registration* Not applicable, as no health care intervention was conducted.

**Supplementary Information:**

The online version contains supplementary material available at 10.1186/s40359-023-01197-0.

## Background

Children and adolescents with mental health problems are psychiatrically hospitalized if outpatient treatment is not sufficient for reducing symptomatology. Inpatient hospitalizations in child and adolescent psychiatry usually last numerous weeks and are generally associated with considerable health improvement [1]. However, between 14 and 38% of psychiatrically hospitalized children and adolescents experience a rehospitalization within 12 months after discharge [2–5], with most readmissions within the first 3 months after discharge [4, 6, 7]. During this period of high risk for rehospitalization, patients face the challenge to adjust to their different post-discharge environments [8] and many demands of the transition after discharge from psychiatric hospitalization are school-related [9, 10]. The need to examine the post-discharge phase closely and interventions to reduce rehospitalizations is accordingly great [11–13]. As reintegration refers to the transition after psychiatric hospitalization to school, the terms reintegration and transition are used synonymously.

### Stressors during transition

Stressors during transition after psychiatric hospitalization are consistently found in the academic, social and emotional domain, all adding to potentially preexisting difficulties [10, 14–16]. Academic stressors concern the risk of patients falling behind at school due to hospitalization [14]. This situation is potentially initiating a vicious circle: the need to catch up the missed work leads to stress, in turn worsening the symptoms, which again impacts learning, and so on [14–16]. The academic situation must be considered specifically in view of mental disorders coming along with a heightened risk for school drop-out and a lower educational attainment over time [17, 18]. In addition to academic stressors, there are social stressors, such as patients reporting problems with peers, bullying, and losing friendships [16, 19]. Social stressors further include not knowing how to handle social situations, being insecure about explaining the personal absence, and concerns about stigmatization [15, 16]. Patients express to be overstrained by social situations, potentially leading to withdrawn behavior and social isolation [14, 16, 19, 20]. Beyond academic and social stressors, emotional stressors exist, and even though mental health usually improves during psychiatric hospitalization, the transition can be a setback in terms of patients’ emotional experiences [14]. Residual symptoms often persist or reappear after discharge, and patients must deal with transition related anxiety, emotional instability, and nervousness [8, 16, 21]. Altogether, patients frequently report to feel emotionally overwhelmed by reentering school, even leading to some patients not fully returning to school [14–16].

In consideration of the stressors in the academic, social, and emotional domain, it is likely that the transition experience is determined by the ability to meet transition demands and to buffer against those stressors [14]. Succeeding at school, having positive relationships and social interactions, and less emotional strain should reduce the amount of stress and thereby positively influence post-discharge adjustment and enhance well-being. Those assumptions are in line with research on primary to secondary school transitions, which shows that good school attendance and increased academic engagement, the ability to build positive and stable peer relationships, as well as control of negative emotions contribute to smooth transitions [22].

### Well-being, self-control, and academic self-efficacy as important variables during transition

Well-being is the affective and cognitive judgment about how well one’s life is going [23–25]. Following the World Health Organization, mental health is defined not only as the absence of symptoms of mental disorders, but also as the presence of well-being [26]. Correspondingly, patient-reported outcomes of subjective health-related quality of life have gained in importance alongside clinical indicators of specific symptoms to assess health care outcomes [27–29]. Low levels of well-being pose a risk factor for future psychopathological symptoms [30, 31], while subjective well-being and psychopathology are predictive of school functioning [32].

Children transitioning from primary to secondary school exhibit a decline in self-control over time, with less decline coming along with better post-transition adjustment [33]. Self-control denotes the capacity to regulate behavior, thoughts, and emotions, allowing to overcome or change dominant response tendencies and is related to a variety of beneficial outcomes [34]. Higher levels of self-control predict better functioning on academic, social, and emotional domains [33], areas in which patients experience vast demands during the transition from psychiatric hospital to school. In the academic domain, higher levels of self-control come along with better attainment at school, in the social domain with less peer problems and better interpersonal relationships as well as in the emotional domain with less emotional problems, better coping with stress and overall better psychological adjustment [33, 35–37].

Regarding the risk of the vicious circle—exacerbating symptoms due to stress arising from catching up the missed schoolwork, in turn impairing learning which again is a stressor—academic self-efficacy likely prevents sliding into the same or helps to break out of it. Academic self-efficacy is the belief about one’s own capability to execute necessary actions that should lead to a desired academic goal [38, 39]. The feeling of competence to master the respective academic situation determines how well a student engages and persists in learning [39]. High academic self-efficacy can be a resource by improving motivation and academic achievement [40, 41], but transitioning from middle to high school comes along with a large decrease of academic self-efficacy [42].

Self-efficacy and self-control should be crucial factors for successful adaptation processes as well as an overall high well-being during reintegration after psychiatric hospitalization. The aim of the present study was to examine patients' well-being during reintegration after psychiatric hospitalization to school as well as patients' self-control and academic self-efficacy as transdiagnostic variables that may enhance patients’ well-being and their ability to cope better with the transition-related stressors.

### Importance of the triadic perspective

Since the individual is not independent of its context [43, 44], the embedding social environments should be considered in addition to individual characteristics of the patient, when investigating the reintegration period. The family and school environments are usually the two most important areas of life for children and adolescents, and mental health problems are often not limited to family settings but also manifest themselves in the school context. From the patient's perspective, their parents and teachers are the most important attachment figures who provide support during reintegration [45].

However, many parents experience high levels of strain during their child's inpatient hospitalization and express concerns about their parenting skills regarding the transition period after discharge [46]. Parental self-efficacy, which is the expectation about the own ability of successful parenting, is associated with parental competence and plays a relevant role for academic, social, and emotional outcomes of a child and its adjustment to school [47–49]. In that light, parental self-efficacy can be an important resource for patients’ recovery and well-being [48], as parents can help their children in coping with demands during reintegration [50]. Parents with lower self-efficacy tend to show less parental involvement [47, 48], in turn being associated with less decrease of symptomatology post-discharge [51] and an increased risk for rehospitalization for the child [6].

Teachers express that they need more knowledge and skills to deal with students returning to school after psychiatric hospitalization, as most students still show problematic behavior in the school context [21]. They report not feeling confident in their ability to manage mental health problems in class [52, 53]. But teachers’ understanding and support is needed by patients during and after psychiatric hospitalization [16, 50]. Teacher self-efficacy is defined as the belief about one’s own capability to influence and support a student’s learning [54]. A high level of self-efficacy among teachers promotes a supportive environment in class, which in turn increases student motivation and academic achievement [55]. Further, teachers with higher levels of teacher self-efficacy are more likely to work persistently and engage positively with challenging students [56, 57]. Additionally, teacher self-efficacy is associated with positive teacher–student-relationships [58], with the quality of these relationships being particularly important for students who are academically at risk [59]. The present study therefore complements patients’ perspectives by the perspectives of parents and teachers for an investigation of this triad and examines self-efficacy among parents and teachers in dealing with the patient as facilitating factors for patients’ transition process after psychiatric hospitalization.

### Present study: investigating between- and within-person processes of patient–parent–teacher triads

Despite a few existing studies [see 10], data on school reintegration after psychiatric hospitalization is limited, particularly for younger children and children outside the United States of America [10]. The current study is, to the best of our knowledge, the first quantitative, intensive longitudinal study applying an ambulatory assessment design to examine patients’ transition to school following psychiatric hospitalization, focusing on patient–parent–teacher triads. It examines patients’ well-being, self-control, and academic self-efficacy, as well as parents’ and teachers’ self-efficacy as important diagnosis-independent variables during the transition. Smartphone-based data of triads were assessed in an intensive longitudinal ambulatory assessment on 50 consecutive school days starting 2 weeks before discharge. Assessing variables in patients’ daily lives enhances generalizability and increases ecological validity while preventing retrospective biases [60–62]. The resulting time series data allow us to investigate within-person processes (variation within an individual) in addition to between-person differences (variations between individuals). Those within-person processes cannot be inferred from the predominant between-person based designs, as evidence from the between-person level cannot validly be transferred to the within-person level [e.g., 63]. Those two levels can only be separated, and hence within-person processes depicted, with an intensive longitudinal study design.

However, it is difficult to control for confounding variables as, for instance, the situations in which questionnaires are answered may differ between assessments [60]. We aimed to attenuate this by assessing and controlling for daily negative and positive events (one item each with a dichotomous response format) as they are found to influence daily well-being [64]. We further followed the prediction-based approach [65] and assessed patients’ pre-discharge well-being, self-control, and academic self-efficacy at baseline to explore if those variables can predict beneficial outcomes during transition. It is plausible that patients with more favorable characteristics have more latitude to deteriorate over time, but at the same time may have more resources buffering against stressors. All those considerations are reflected in our hypotheses formulated below.

#### **Hypothesis 1**

Time trend effects.

We expected patients’ well-being (H1a), self-control (H1b), and academic self-efficacy (H1c) to decline on average over time during the transition period. We further expected patients to differ significantly in this trend over time. We aimed to explore if the individual specific time-effect for each variable is associated with the respective extent of well-being (H1aa), self-control (H1ba), and academic self-efficacy (H1ca) at baseline, whereby both an attenuating or boosting effect coming along with higher levels is conceivable.

#### **Hypothesis 2**

Between-person effects of the patients and triads.

We expected patients generally showing higher self-control (H2a) and academic self-efficacy (H2b) to also show higher levels of well-being. Further, we expected patients with parents (H2c) and teachers (H2d) of generally higher parental or teacher self-efficacy regarding the child to show higher levels of well-being.

#### **Hypothesis 3**

Within-person effects of the patients.

We expected higher well-being on days with higher self-control (H3a) and higher academic self-efficacy (H3b). That is, we expected structural dependence on the within-person level of patients’ well-being and self-control as well as academic self-efficacy.

#### **Hypothesis 4**

Within-person effects of the triads.

We expected higher patient well-being on days with higher parental self-efficacy (H4a) and on days with higher teacher self-efficacy (H4b). That is, we expected structural dependence on the within-person level of patients’ well-being and parents’ parental self-efficacy as well as teachers’ teacher self-efficacy.

## Methods

### Design and aim

The present study is an intensive longitudinal study applying an ambulatory assessment design to examine patients’ transition to school after psychiatric hospitalization, focusing on patient–parent–teacher triads. It aims to examine patients’ well-being, self-control, and academic self-efficacy, as well as parents’ and teachers’ self-efficacy as important diagnosis-independent variables during the transition.

### Participants

Participants were recruited from 2016 to 2018 at the psychiatric day hospital for children in the Department of Child and Adolescent Psychiatry, Psychosomatics, and Psychotherapy of the University Hospital Tuebingen, Germany. During their treatment, clinic employees informed them about the study. Exclusion criteria were inability to attend school, a profound developmental disorder without language development or a psychotic disorder. A total of 27 children with belonging 24 parents and 20 teachers were recruited. Two children dropped out during the study, resulting in the final sample of 25 children, ranging in age from 7 to 13 years (*M* = 10.58, *SD* = 1.62). Total sample description is displayed in Table 1, with primary diagnosis based on the German edition of 10th Revision of the Classification of Mental and Behavioral Disorders (ICD-10, [66]). Patients and parents received a cinema coupon compensation, with monetary amount ranging from 10 Euro (first 20 days participation) over 20 Euro (first 40 days participation) to 25 Euro (50 days participation). Teachers got compensated with a book present.

**Table 1:** Sample characteristics

	*N* (%)
Gender	
Female	10 (40)
Male	15 (60)
Type of school	
Primary school	14 (56)
Special education school	2 (8)
Secondary school	9 (36)
Primary diagnoses (ICD-10)	
Mood (affective) disorders (F30-F39)	2 (8)
Neurotic, stress-related and, somatoform disorders (F40-F48)	4 (16)
Behavioral syndromes associated with physiological disturbances and physical factors (F50-F59)	2 (8)
Disorders of psychological development (F80-F89)	2 (8)
Behavioral and emotional disorders with onset usually occurring in childhood and adolescence (F90-F98)	15 (60)

### Material

The present study is part of a larger one that examined environment-related predictors of successful day hospital treatment [67]. As we focused on the ambulatory assessment data related to the formulated hypotheses in the present study, only the measures used for these analyses are presented in detail.

#### Baseline measures

*Well-being* For the assessment of well-being, we used the emotional well-being subscale of the revised, self-report version for children aged 7 to 13 of the questionnaire for measuring health-related quality of life (Kid-KINDL^R^, [68, 69]). The subscale consists of four items asking for the frequency of events during the last week with response categories ranging from “never” to “all the time” on a five-point Likert-Scale (e.g., “During the past week, I had fun and laughed a lot.”) with literature evidence of satisfying reliability (Cronbach’s α = 0.68) and validity [68, 69]. For the current study, it also resulted in a still satisfying reliability (Cronbach’s α = 0.50).

*Self-control* For the assessment of self-control, we used the brief German parent-rating version of the Self-Control Scale (SCS-K-D, [70]). We adapted the 13 items by simplifying the language to be suited as a self-report questionnaire for children, also resulting in satisfying internal consistency for the current study (Cronbach’s α = 0.84). Patients had to rate the extent to which the statements apply to them in general, from “not at all” to “totally true” on a five-point Likert scale (e.g., “I do nothing that I will regret later.”).

*Academic self-efficacy* For the assessment of academic self-efficacy, we used an established scale consisting of seven items (WIRKSCHUL, [71]). We adapted the items by simplifying the language for children, also resulting in satisfying internal consistency for the current study (Cronbach’s α = 0.67). Patients had to rate the extent to which the statements apply to them in general, from “not at all” to “totally true” on a five-point Likert scale (e.g., “I can solve even complex tasks in class, when I make an effort.“).

#### App-based daily measures on the smartphone

The application used for the ambulatory assessment was movisensXS [72] running on the NEXUS 5 smartphone by LG Electronics, provided for all participants. For a complete overview of all daily items see Additional file 1 – Daily Measures.

*Well-being* For the daily assessment of well-being, we developed one item globally asking for how they were overall today (“How were you overall today?”). This is in accordance with just slightly differing single-items asking for well-being, exhibiting satisfying validity and reliability in the literature [24, 73–75]. Possible answers ranged from “very bad” to “very good” on a five-point Likert scale.

*Self-control and academic self-efficacy* For the daily assessment of self-control and academic self-efficacy, we shortened the scales used at baseline (i.e. an adaption of the brief German parent-rating version of the Self-Control Scale, SCS-K-D, [70] and the scale WIRKSCHUL, [71]) to four and three items, respectively. For an overview of all items see Additional file 1 – Daily Measures (e.g. self-control, “Today, I have done something, I regretted later”; e.g. academic self-efficacy, “Today, I was able to solve even complex tasks in class, when I made an effort”). Further, we changed the referring time frame from general to the present day. The reliabilities (Cronbach’s α and McDonald’s ω) for the current study are reported in Table 3 in the results section.

*Negative and positive events* To control for daily negative and positive events, children were asked what happened at school with one item each (“Did something happened today that you thought was bad/good at school?”) and a dichotomous response format (yes/no). Following a positive response, participants were able to indicate what exactly happened to them in a free answer format (“If something like that happened today, what was it?”).

*Parental and teacher self-efficacy* For the assessment of parental and teacher self-efficacy, we used three items of an established scale (WIRKLEHR, [76]). We changed the referring time frame from general to the present day. Items further got appropriately adapted to fit the reference to the specific child (i.e., the/my child; e.g., parent: “Today I was able to guide my child also in problematic situations.”; teacher: “Today I was convinced that I can teach the child the subject material also in problematic situations.”). The reliabilities (Cronbach’s α and McDonald’s ω) for the current study are reported in Table 3 in the results section.

### Procedure

**Fig. 1:**
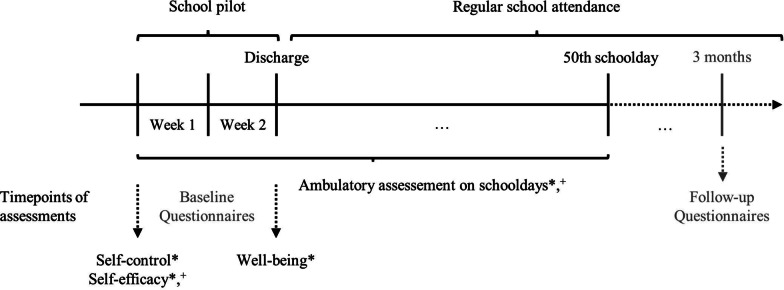
Overview of assessment procedure with * assessed from patients and ^+^ assessed from parents and teachers

An overview of the procedure can be seen in Fig. 1. During the patients’ stay at the psychiatric hospital, the patients and parents were informed about the study by an employee of the day hospital. In case of general interest in participation, the patients, and their parents, as well as a teacher of the patient, were invited to the study. All subsequent study appointments were held by study employees of the psychiatric hospital. Verbal and written study information, including informed consent forms, was provided for parents and patients, after a regular appointment at the psychiatric hospital. For teachers, this information appointment took place either by phone and post or at the hospital.

After the participants gave written informed consent, an introduction appointment was made, separately for the patients with parents and the teacher, one or two days before the school pilot period started. All participants filled out baseline measure questionnaires (only patients’ well-being was assessed at discharge, as being part of standard diagnostic, to minimize patient’s burden). A smartphone was delivered to all participants, and they were introduced to the application implementation. A smartphone-based measurement burst for 50 consecutive school days was conducted, starting with the first day of regular school attendance of the school pilot period. The school pilot period is a phase around 2 weeks before discharge, where patients attend their regular school but subsequently continue to attend the psychiatric hospital. If the school pilot period proceeds successfully, patients get discharged.

Participants were prompted with an acoustic signal at half past six in the evening to answer the smartphone-based measurement questions. It was possible to work on the questions self-initiated between five and nine o’clock in the evening. Introduction and items were presented audiovisually, so that children who were not yet confident readers could have the items read aloud. Questions with free answer format could be answered and saved by voice recording. This daily ambulatory assessment took about five minutes per day. Data got transferred from the smartphone and safely stored on the servers of the university and university hospital during the routine appointments at follow-up. The final appointment was further used for all participants to fill out follow-up questionnaires [67].

### Data analyses

Multilevel modeling analyses with repeated measurements (Level 1) nested within patients (Level 2) were calculated to account for the multilevel structure of ambulatory assessment data [77]. Data was analyzed with the statistical software R [78]. We calculated multilevel models with the “nlme” package [79]. Multilevel reliability was calculated with the “reliability” function of the “semTools” package [80]. We centered the predictors on the grand mean for between-person effects and on the personal mean for the within-person effects. An overview of all equations is depicted in Additional file 2 – Mixed models equations. We depicted the most complex model below for description of model building, whereby simpler models arise by leaving out respective predictors.

That is, for the depicted model, we investigated *well-being*_ij_ as outcome measure for individual i (i = 1, …, *N*) on day j (j = 1, …, n_i_). We stepwise built on the unconditional random-intercept only model, with firstly introducing the two dummy-coded (0 = no, 1 = yes), fixed time-varying level 1 predictors of *negative* (γ_10_) and *positive events* (γ_20_). Then, we added the fixed time-varying level 1 predictor *day* (γ_30_)*,* which was next allowed to vary between participants (μ_3i_). We then introduced the fixed time-varying level-1 predictors (γ_40,_ γ_50_), being personal mean centered, giving insight into within-person relationships, which are allowed to vary randomly (μ_4i,_ μ_5i_). After specifying all level-1 predictors, we added the fixed time-constant, grand-mean centered level-2 predictors (γ_01,_ γ_02_), giving insight into between-person relationships. Hence, the intercept (β_0i_) of each patient is modeled as a function of the mean intercept (γ_00_ + γ_01_(between_i_) + γ_02_(between_i_)) and random error (μ_0j_). The slopes (β_1i,_ β_2i,_ β_3i,_ β_4i,_ β_5i_) as a function of the mean slope (γ_10,_ γ_20,_ γ_30,_ γ_40,_ γ_50_) and respective random error (μ_3i,_ μ_4i,_ μ_5i_; [77]). All variables, up to control variables, were included if significant.

Altogether, model 1 includes the trend of well-being (H1a), and its’ relationship with patients’ self-control and academic self-efficacy as within- and between-person effect, controlled for daily events. Model 2 and model 3 include the trend of self-control and academic self-efficacy of the patient. Model 4 and model 5 include the relationship between patients’ well-being and parental respective teacher self-efficacy as within- and between-person effect, controlled for time and daily events. We separated the model for patients’ and triads’ variables due to huge differences in compliance for patients, parents, and teachers, resulting in different amount of missing data which results in model non-convergence if not separated.

Equation for m1:

$${\text{Level}}\;1\;{\text{well - being}}_{{{\text{ij}}}} =\upbeta _{{0{\text{i}}}} +\upbeta _{{1{\text{i}}}} ({\text{negative}}\;{\text{event}}_{{{\text{ij}}}} ) +\upbeta _{{2{\text{i}}}} ({\text{positive}}\;{\text{event}}_{{{\text{ij}}}} {)} +\upbeta _{{3{\text{i}}}} ({\text{day}}_{{{\text{ij}}}} ) +\upbeta _{{4{\text{i}}}} ({\text{within self - control}}_{{{\text{ij}}}} ) +\upbeta _{{5{\text{i}}}} ({\text{within}}\;{\text{academic}}\;{\text{self - efficacy}}_{{{\text{ij}}}} ) + r_{{{\text{ij}}}}$$

$$\begin{array}{*{20}l} {{\text{Level}}\;2} \hfill & {\upbeta _{{0{\text{i}}}} =\upgamma _{00} +\upgamma _{01} ({\text{between}}\;{\text{self - control}}_{{\text{i}}} ) +\upgamma _{02} ({\text{between}}\;{\text{academic}}\;{\text{self - efficacy}}_{{\text{i}}} ) +\upmu _{{0{\text{i}}}} } \hfill \\ {} \hfill & {\upbeta _{{1{\text{i}}}} =\upgamma _{10} } \hfill \\ {} \hfill & {\upbeta _{{2{\text{i}}}} =\upgamma _{20} } \hfill \\ {} \hfill & {\upbeta _{{{\text{3i}}}} =\upgamma _{30} +\upmu _{{{\text{3i}}}} } \hfill \\ {} \hfill & {\upbeta _{{{\text{4i}}}} =\upgamma _{40} +\upmu _{{{\text{4i}}}} } \hfill \\ {} \hfill & {\upbeta _{{{\text{5i}}}} =\upgamma _{50} +\upmu _{{{\text{5i}}}} } \hfill \\ \end{array}$$

Correlations between random effects were calculated, except if the model did not reach convergence. Fixed and random effects were tested with likelihood ratio test comparing the appropriate (nested) models, and when found to be significant, predictors were added to the model. To account for correlation of adjacent time points, we applied the first-order autoregressive error structure (AR(1), [81]). In case of time slopes varying randomly, we calculated bivariate correlations using Pearson’s *r* between the individual specific trend over time and the corresponding (grand-mean centered) baseline measure, to find out if there is a relationship between baseline measures and individual slopes. All tests assumed a significance level of α = 0.05.

## Results

### Compliance

We calculated compliance rates as the percentage of prompts being responded to, averaged over participants (potential maximum: patients 50 × 25 = 1250, parents 50 × 24 = 1200, teachers 50 × 20 = 1000). The mean compliance rate for each of patients’ variables was 71%, except for positive events resulting in 70% (*SD* = 19%, range = 18–94, *N*_well-being/self-efficacy/negative event_ = 882, *N*_self-control_ = 883, *N*_positive event_ = 873). Compliance rates for the specification of daily events are calculated as percentage of given free answers in case of the occurrence of an event. These compliance rates are on average 80% (*SD* = 34%, range = 36–100, *N* = 108) for negative ones and 87% (*SD* = 22%, range = 33–100, *N* = 335) for positive ones. For parents, the compliance rate was 72% (*N* = 866, *M* = 72%, *SD* = 22%, range = 16–98), and for teachers 43% (*N* = 427, *M* = 43%, *SD* = 20%, range = 2–70).

### Descriptive statistics

**Fig. 2:**
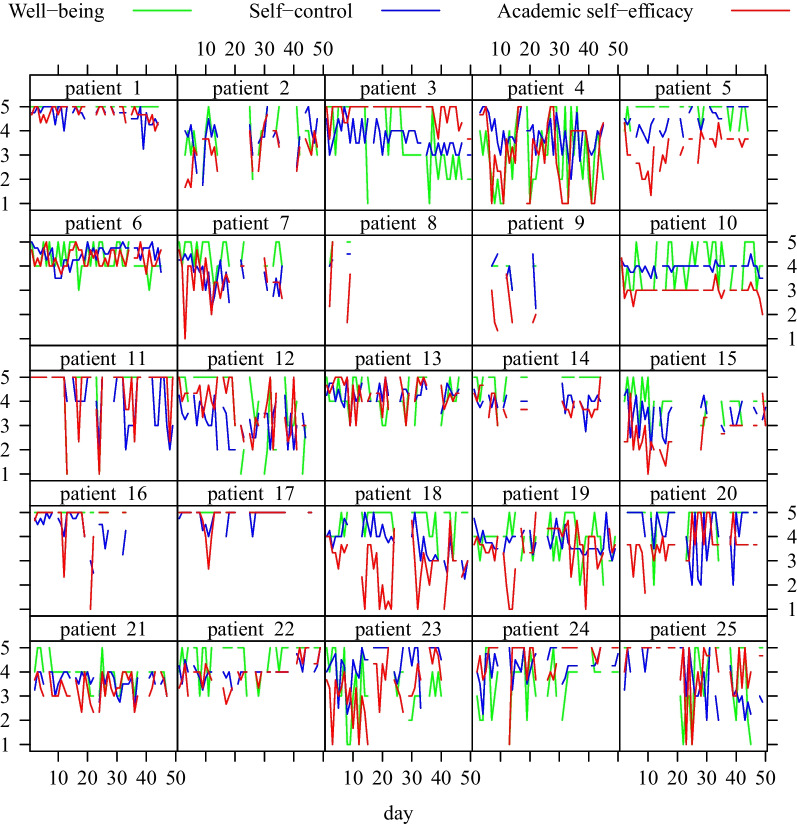
Answers over time of well-being, self-control, and academic self-efficacy, separated by patients

Descriptive statistics for the baseline measures are provided in Table 2. We computed reliability at each level by using multilevel confirmatory factor analysis [82], with Cronbach’s alpha (α) and McDonald’s omega (ω) depicted in Table 3. Intraclass correlations (ICCs) were computed, that is the ratio of the between person variance to the total variance, indicating if multilevel analysis is adequate [77]. Within-person variability is indicated by the intra-individual standard deviation (ISD), a measure for the amplitude of fluctuation [83]. Due to the longitudinal design, we further considered the mean square successive difference (MSSD), additionally taking the temporal dependency into account, with higher scores displaying higher degrees of instability [60, 83, 84]. This is important as individuals with the same ISDs can exhibit different MSSDs due to different amount of temporal dependency [83]. Descriptive statistics as well as ICCs, ISDs, and MSSDs are depicted in Table 4. The answers over time of the patient’s variables can be seen in Fig. 2.

**Table 2:** Descriptive statistics of baseline measures for well-being, self-control, and academic self-efficacy

Variable	*M *(*SD*)	Range
Well-being	4.19 (0.52)	3.24–5.00
Self-control	3.03 (0.31)	2.39–3.62
Academic self-efficacy	3.51 (0.64)	1.57–4.57

Assessed by using Kid-KINDL^R^, SCS-K-D, WIRKSCHUL with five-point Likert scale response format ranging from 1 (not at all/never) to 5 (totally true/all the time)

**Table 3:** Within- and between-person reliability for the daily measures

	Self-control	Academic self-efficacy	Parental self-efficacy	Teacher self-efficacy
*Within*				
α	0.505	0.719	0.883	0.739
ω	0.518	0.731	0.886	0.752
*Between*				
α	0.518	0.806	0.968	0.815
ω	0.583	0.813	0.968	0.850

Calculated with Cronbach’s alpha (α) and McDonald’s omega (ω)

**Table 4:** Descriptive statistics for the daily assessed variables

Variable	*M *(*SD*)	Range	ICC	ISD	MSSD
				*M *(*SD*)	*M *(*SD*)
Well-being	4.26 (0.54)	1.00–5.00	0.27	0.73 (0.39)	0.82 (0.70)
Self-control	4.03 (0.41)	1.75–5.00	0.28	0.59 (0.24)	0.47 (0.35)
Academic self-efficacy	3.74 (0.74)	1.00–5.00	0.43	0.77 (0.33)	0.79 (0.60)
Parental self-efficacy	3.76 (0.65)	1.00–5.00	0.56	0.57 (0.24)	0.51 (0.36)
Teacher self-efficacy	4.06 (0.46)	2.33–5.00	0.46	0.47 (0.13)	0.18 (0.17)
Negative event	0.15 (0.14)	0.00–0.50	0.12	0.29 (0.19)	0.16 (0.14)
Positive event	0.46 (0.33)	0.02–1.00	0.41	0.37 (0.13)	0.20 (0.14)

Five-point Likert scale response format ranging from 1 (very bad/not at all) to 5 (very good/totally true), except for negative/positive event, being dummy-coded with 0 (no) and 1 (yes) which is presented as percentage of occurrence over days. Intraclass correlations (ICC), intra-individual standard deviation (ISD), mean square successive difference (MSSD)

### Multilevel analyses

To examine the assumption that data are missing at random, we investigated the relationship between missingness and baseline measures, average daily measures, gender, age, and IQ. There were no interrelations, hence, missingness of data can be ignored [85].

Final models of the multilevel analyses are depicted in Tables 5, 6 and 7. A complete overview of all models is depicted in Additional file 3 – Multilevel analyses. The models m1, m4, and m5 all include well-being as the dependent variable and time as the predictor variable, which is reported only for the first time in the following for reasons of clarity. This is justified as this effect did not differ in significance and only slightly in extent.

**Table 5:** Results of mixed model analysis, well-being depending on daily events, time, self-control, and academic self-efficacy

	Well-being (m1)
*Fixed effects*	
Intercept	4.397 (0.116)
Negative event	− 0.474* (0.077)
Positive event	0.149* (0.065)
Day	− 0.007* (0.004)
SC_within_	0.178* (0.064)
SE_within_	0.207* (0.057)
SC_between_	0.602* (0.165)
*Random effects*	
Intercept	0.476
Day	0.016*
SC_within_	0.211*
SE_within_	0.206*
Residual	0.724
*logLik*	− 1005.494
AR(1)	0.122

Depicted are estimates (standard error) of fixed effects and random effects as standard deviations, *p < .05. Description of variables: SC = self-control, SE = academic self-efficacy, negative/positive event dummy-coded (0 = no, 1 = yes). *N*_Level 2_ = 25, *N*_Level 1_ = 872

**Table 6:** Results of mixed model analyses, self-control, and academic self-efficacy depending on time

	Self-control (m2)	Academic self-efficacy (m3)
*Fixed effects*		
Intercept	4.198 (0.101)	3.674 (0.178)
Day	− 0.008* (0.004)	0.004 (0.004)
*Random effects*		
Intercept	0.459	0.833
Day	0.018*	0.014*
Residual	0.579	0.785
*logLik*	− 817.826	− 1066.239
AR(1)	0.137	0.231

Depicted are estimates (standard error) of fixed effects and random effects as standard deviations, *p < .05. m2: *N*_Level 2_ = 25, *N*_Level 1_ = 883; m3: *N*_Level 2_ = 25, *N*_Level 1_ = 882

**Table 7:** Results of mixed model analyses

	Well-being (m4)		Well-being (m5)
*Fixed effects*		*Fixed effects*	
Intercept	4.384 (0.119)	Intercept	4.427 (0.140)
Negative event	− 0.679* (0.092)	Negative event	− 0.611* (0.135)
Positive event	0.209* (0.079)	Positive event	0.409* (0.113)
Day	− 0.006* (0.004)	Day	− 0.009* (0.006)
SE_p,within_	0.138* (0.051)		
*Random effects*		*Random effects*	
Intercept	0.428	Intercept	0.358
Day	0.017*	Day	0.017*
Residual	0.809	Residual	0.755
*logLik*	− 940.384	*logLik*	− 369.099
AR(1)	0.162	AR(1)	0.156

Well-being depending on daily events, time, and parental (m4) respective teacher (m5) self-efficacy. Depicted are estimates (standard error) of fixed effects and random effects as standard deviations, * *p* < .05. Description of variables: SE_p_ = parental self-efficacy, SE_t_ = teacher self-efficacy, Negative/ positive event dummy-coded (0 = no, 1 = yes). m4: *N*_Level 2_ = 24, *N*_Level 1_ = 756; m5: *N*_Level 2_ = 20, *N*_Level 1_ = 307

*Hypothesis* 1*: Time trend effects. *We expected patients’ well-being (H1a), self-control (H1b), and academic self-efficacy (H1c) to decline on average over time during the transition period. We further expected patients to differ significantly in this trend over time. We aimed to explore if the individual specific time-effect for each variable is associated with the respective extent of well-being (H1aa), self-control (H1ba), and academic self-efficacy (H1ca) at baseline, whereby both an attenuating or boosting effect coming along with higher levels is conceivable.

**Fig. 3:**
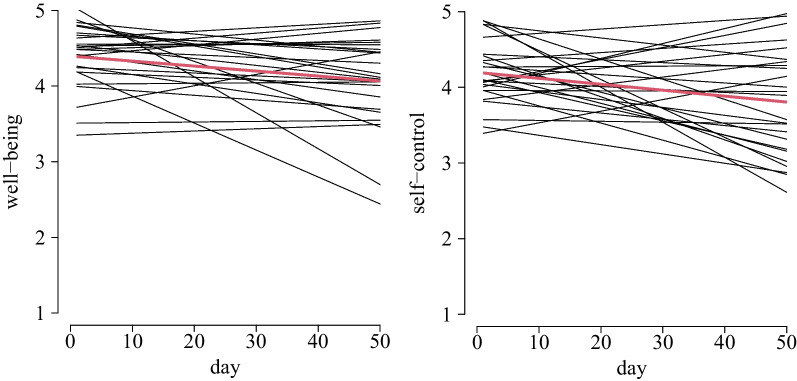
Estimated mean trend over time (red) and individual specific effects of well-being (left) and self-control (right)

Patients’ well-being significantly declined over time on average (m1: γ_30_ = − 0.007, *SE* = 0.004; H1a), that is, over 50 school days, it decreased about 0.35 points. The trend over time differed significantly between patients (m1: σ_μ3j_ = 0.016; Fig. 3). Patients’ self-control significantly declined over time on average (m2: γ_10_ = − 0.008, *SE* = 0.004; H1b), that is, over 50 school days, it decreased about 0.40 points. The trend over time differed significantly between patients (m2: σ_μ1j_ = 0.018; Fig. 3). Patients’ academic self-efficacy did not decline over time (m3: γ_10_ = 0.004, *SE* = 0.004; H1c), but patients differed significantly with regards to their trend over time (m3: σ_μ1j_ = 0.014).

There was neither a significant correlation between baseline well-being and individual specific trends over time of well-being (*r*(23) = 0.20, *p* = 0.344; H1aa), nor between baseline self-control and individual specific trends over time of self-control (*r*(21) = − 0.19, *p* = 0.398; H1ba), nor between baseline academic self-efficacy and individual specific trends over time of academic self-efficacy (*r*(21) = − 0.19, *p* = 0.398; H1ca).

*Hypothesis* 2*: Between-person effects of the patients and triads. *We expected patients generally showing higher self-control (H2a) and academic self-efficacy (H2b) to also show higher levels of well-being. Further, we expected patients with parents (H2c) and teachers (H2d) of generally higher parental or teacher self-efficacy regarding the child to show higher levels of well-being.

On a between-person level, there was a significant positive relationship between patients’ well-being and self-control (m1: γ_01_ = 0.602, *SE* = 0.165; H2a), meaning that children with one point more self-control than the average child experience a higher well-being of about 0.602 than the average child. There was no significant relationship between academic self-efficacy (m1: γ_02_ = 0.119, *SE* = 0.108; H2b), parental self-efficacy (m4: γ_01_ = 0.063, *SE* = 0.145; H2c), or teacher self-efficacy (m5: γ_01_ = 0.376, *SE* = 0.215; H2d) and patients’ well-being.

*Hypothesis* 3*: Within-person effects of the patients. *We expected higher well-being on days with higher self-control (H3a) and higher academic self-efficacy (H3b). That is, we expected structural dependence on the within-person level of patients’ well-being and self-control as well as academic self-efficacy.

**Fig. 4:**
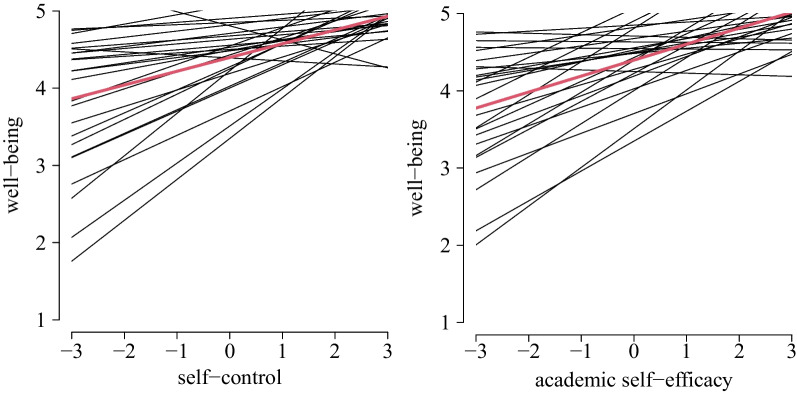
Estimated within-person relationship between self-control and well-being (left) and academic self-efficacy and well-being (right) on average (red) with individual specific effects

There was a significant positive within-person relationship between well-being and self-control (m1: γ_40_ = 0.178, *SE* = 0.064; H3a), meaning that on days with an increase of one point of self-control compared to the personal mean, well-being increases about 0.178. The relationship significantly differed between patients (m1: σ_μ4j_ = 0.211; Fig. 4). There was a significant positive within-person relationship between well-being and academic self-efficacy (m1: γ_50_ = 0.207, *SE* = 0.057; H3b), meaning that on days with an increase of one point of academic self-efficacy compared to the personal mean, well-being increases about 0.207. The relationship significantly differed between patients (m1: σ_μ5j_ = 0.206; Fig. 4).

*Hypothesis* 4: *Within-person effects of the triads.* We expected higher patient’s well-being on days with higher parental self-efficacy (H4a) and on days with higher teacher self-efficacy (H4b). That is, we expected structural dependence on the within-person level of patients’ well-being and parents’ parental self-efficacy as well as teachers’ teacher self-efficacy.

**Fig. 5:**
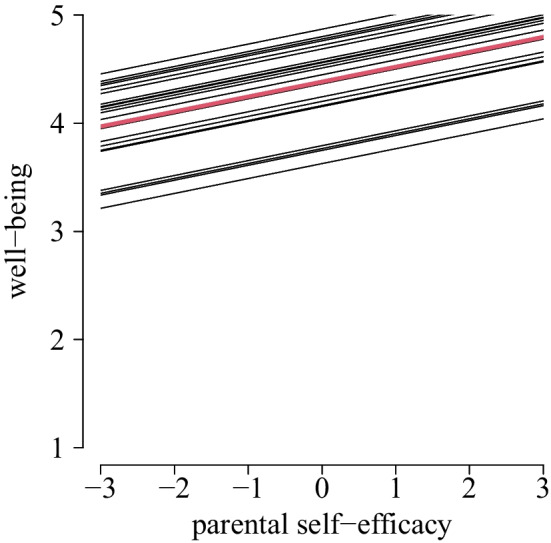
Estimated within-person relationship between parental self-efficacy and well-being on average (red) with individual specific effects

There was a significant positive within-person relationship between patients’ well-being and self-efficacy of the parent (m4: γ_40_ = 0.138, *SE* = 0.051; H4a), meaning that on days with an increase of one point of parental self-efficacy compared to the personal mean, patients’ well-being increases about 0.138. The relationship is not different between patients (m4: σ_μ4j_ = 0.192; Fig. 5). There was no within-person relationship between patients’ well-being and self-efficacy of the teacher (m5: γ_40_ = 0.146, *SE* = 0.096; H4b), further not differing between patients (m5: σ_μ4j_ = 0.001).

### Qualitative reports of daily events

The statement whether a positive or negative event occurred, served as a control variable with a dichotomous response format (yes/no). In case of a yes-response, the event could be specified in a free answer format. Although these events were not the focus of the present study, qualitative reports provide insights and therefore some exemplary responses on positive and negative events are given in the following. Among negative events, patients reported, for example, not being able to be attentive, having difficult exercises and too much homework, receiving bad grades, having social conflicts with peers and teachers, experiencing bullying, and somatic symptoms such as abdominal pain or headache. Among positive events, patients reported, for example, less difficult tasks and less homework, receiving good grades, being able to participate in class, having positive social contacts with peers, and getting praised from teachers.

## Discussion

The present study investigated between- and within-person processes of patient–parents–teacher triads during transition from psychiatric hospitalization to school. The results show that over 50 consecutive schooldays, patients’ well-being and self-control decreased on average over time (H1a, H1b). Even though patients’ academic self-efficacy did not decrease on average (H1c), patients experienced instability and fluctuation of their self-efficacy over time. There was no significant relationship between the individual specific trends over time and the extent of the same variables before the transition (H1aa, H1ba, H2ca). At the between-person level, patients with general higher self-control experienced general higher well-being (H2a). However, there was no significant between-person relationship between patients’ well-being and patients’ academic self-efficacy (H2b), parents’ (H3c) or teachers’ self-efficacy (H3d). Importantly at the within-person level, patients experienced higher well-being on days with higher self-control and academic self-efficacy (H3a, H3b). Further patients experienced higher well-being on days with higher parental self-efficacy (H4a), but not teacher self-efficacy (H4b).

The present results, especially the decrease of patients’ well-being and self-control in the weeks following discharge, emphasize that reintegration can be very challenging for patients. Moreover, lower well-being increases the risk for psychopathological symptoms [30, 31] and, in adults, decreases the chance for recovering [86]. The present findings are therefore consistent with studies showing that the period immediately after discharge is associated with a heightened risk for rehospitalization [4, 6, 7]. It seems reasonable to assume that causes for the decrease of patients’ well-being are comparable to the challenges patients have reported in other studies during reintegration, such as having to make-up schoolwork or experiences of exclusion [e.g., 14, 16, 19. Evidence for these challenges can also be found in the present qualitative results regarding negative events at school. For example, in the present study, patients reported academic difficulties such as poor performance as well as social difficulties such as bullying and conflicts with peers during the transition period after discharge. Patients’ self-control also declined over the course of the psychiatric hospital to school transition, as already shown in the context of school-to-school transitions [33]. Transition-related stressors are again a likely explanation for the decline in patients’ self-control, as an increase in stress over middle childhood prospectively predicts a decrease in self-control [87]. Interestingly, patients’ academic self-efficacy did not exhibit a decline over time. As most patients attended primary school, academic demands may be easier to meet, thereby perhaps not undermining academic self-efficacy as much. Nevertheless, it is very positive that patients’ academic self-efficacy did not decline during transition and may form a resource that patients can draw on to meet the accompanying academic demands.

Beyond the average developments, patients differed meaningfully concerning the individual trend in well-being, self-control, and academic self-efficacy over time, being in accordance with individually different reports concerning school reentry [16]. A few patients in the present study even showed a positive development during reintegration, which can be seen in the individual specific effects of well-being showing positive slopes in Fig. 3 in the results section. This makes it conceivable that there exist factors promoting a successful transition. Identifying factors promoting a successful transition by specifically looking at patients with positive transition developments could yield insights for promising future interventions to provide greater assistance to patients at risk for negative developments. For example, as concerns for emotions when considering returning to school, as well as psychological and emotional difficulties pre-discharge, come along with a less favorable post-discharge experience [16], these factors should be considered in future studies. One aim of the present study was to explain heterogeneity with pre-discharge factors to predict individual specific trends over time. However, patients’ well-being, self-control and academic self-efficacy did not turn out to be relevant predictors. Our daily measurements show that these constructs are subject to considerable fluctuations. Hence, a single measurement at baseline being only an extract can be a reason for the missing effect, underlining the importance of repeated longitudinal measurements.

In the present study, it turned out that self-control and academic self-efficacy can be important strengths for patients to draw upon, as on days with higher levels of self-control and academic self-efficacy, patients experienced higher well-being. This relationship is existent beyond the influence of daily events. We cannot claim definitive answers about causality, but it seems plausible that on days with higher self-control and academic self-efficacy, patients are more capable to cope with academic, social, and emotional demands in the transition period. That is, patients may be more likely to make up the missed schoolwork [39], have positive interactions with others and a better psychological adjustment [35]. By that, adaption to post-discharge environments should be facilitated. This is in line with findings from school-to-school transitions, showing that the ability to control negative emotions and good school attendance can be protective factors against negative impacts of the transition [22].

The present study further showed that on days with higher parental self-efficacy, patients experienced higher well-being. It is very likely that parents will be able to support the child more consistently and aid to buffer against the number of stressors the child is faced with on days with higher parental self-efficacy. Further, supporting, engaging and responsive parents are found to be a resource for positive school-to-school transitions [22]. As teacher self-efficacy [see 55 for a review] was assumed to be important for patients during transition, it is surprising to not be a significant predictor of patients’ well-being. However, as teachers’ compliance rate was rather low, and a trend in the expected direction was evident, we cannot make a concluding statement and more research is needed on this matter.

Looking at the comparison between the within- and between-person level, we found that patients with generally higher self-control generally report higher well-being. Except for this effect, we did not find a significant relationship on the between-person level for well-being and academic, parental, and teacher self-efficacy. This contrasts with the positive within-person effects between well-being and academic and parental self-efficacy. Those results underline that aggregated data does not represent the individual, and effects on the group and individual levels are not implicitly related [88]. Hence, research being based on the group level informing about practical implications for treatment can be problematic, as between-person findings cannot be generalized to the individual [89]. By solely looking at between-person relationships, we would not conclude academic self-efficacy and parental self-efficacy to be important for the transition period. However, within-person processes reveal that those two are promising to support patients.

### Implications

Implications of the present results include different ways patients can be supported in the transition period aiming not only high but also stable levels of well-being. This means, for instance, strengthening patients’ self-control [see e.g. 90, 91], and academic self-efficacy [see e.g. 92, 93], but also parents’ self-efficacy [see e.g. 47, 94–96], through interventions during treatment and aftercare. Well-evaluated aftercare programs are needed to help patients, as well as their relevant attachment figures, to cope with stressors and occurring problems for a successful reintegration and to stabilize their well-being. As the relationships between well-being and self-control or academic self-efficacy differ between patients, with a few also showing negative relationships, future studies should aim at gaining a deeper understanding by investigating possible moderators of those relationships. It is very likely that well-being is fueled by multiple sources, with the ones assessed in the present study being important ones among others. We aimed to control for daily events to find interrelations of well-being and other variables independent of external events. However, as daily events are significantly related to daily well-being [64], specifying them may reveal important contributors of well-being. That is, good grades, receiving praise of the teacher and being able to participate in class came along with an increase of daily well-being. On the other hand, not being able to concentrate, numerous and complex exercises and receiving bad grades came along with a decrease of daily well-being. Hence, the ability to cope with academic situations seems to have a major impact on patients’ well-being, as also evidenced by the positive within-person relationship between academic self-efficacy and well-being. Thus, attachment figures or mental health professionals talking with the patient about negative events and promoting positive ones seem helpful, emphasizing the importance of supportive accompaniment and aftercare during the transition from psychiatric hospitalization to school. Furthermore, future studies may expand the number of perspectives by including patients’ peers, as patients are concerned about reactions of their peers to their return to school as well as effects of their absence on friendships [8, 14]. In the light of the current COVID-19 pandemic, it can be additionally surmised, that the frequent transition between schooling contexts from home to school, could be associated with partially similar challenges such as concerns about friendships due to the social distancing, making up schoolwork or emotional re-adjustment at school.

### Limitations

For the app-based daily measures on smartphones, established scales had to be shortened to minimize the study's burden on families. This resulted, however, in rather low reliability only for the self-control scale from daily assessment and underlines the need to develop short questionnaires, which are even more important for strained samples. For the assessment of self-efficacy in a daily manner, we decided to use both items for self-efficacy and self-efficacy related experiences, expecting experiences to fluctuate more from day to day. Even though not being established, this is warranted by the internal consistency of the resulting scale. Further, it is an advantage of ambulatory assessment to ask for situations of the present day, as the way individuals master single situations builds one of the most important sources for self-efficacy [97–100]. However, the operational definition of this study, asking for past situations of the present day, therefore shows a discrepancy with the theoretical definition. This should be considered when interpreting the results.

Further, well-being was assessed as part of the standard diagnostic at discharge and thus approximately 2 weeks later compared to the other two baseline measures of self-control and self-efficacy. However, as none of the variables proved to be a relevant predictor of the individual specific trends over time, this temporal offset at baseline seems negligible.

Despite the satisfying compliance rates, which are comparable to other studies [see 101, 102 for reviews], not all children treated at the day hospital during the 2-year recruitment period participated in the present study, which must be considered when making statements about the generalizability of the present results. For all participants, answering daily questions was rather time-consuming, which was probably a barrier to study participation. Reasons for the still satisfying compliance rates, particularly regarding the 50-day study length, may be the graduated reward, the feeling of being taken seriously with one's situation through daily questioning, and the automatic repeated prompts, which increase compliance rates especially in clinical samples [102]. However, teachers' compliance rates were comparatively much lower than those of patients and parents. This may be because rewards for teachers were not graduated, and daily questioning took part in the evening, falling in the end of work, as well as because teachers were not affected by the patients’ transition to the same extent. The low compliance rates among teachers may be one reason that teacher self-efficacy was not a significant predictor of patients’ well-being.

The statement whether a positive or negative event occurred, served as a control variable with a dichotomous response format. Furthermore, the level of distress caused by a negative event could be predictive of daily well-being. However, since we had to limit the number of daily questions so that the burden on patients did not become too high and since this was only a control variable, distress was not assessed additionally. In future studies, this aspect can possibly be considered further. Future studies can also further examine between- and within-person processes during reintegration using additional methods of analysis (e.g., diary analysis using hierarchical linear modeling or latent growth curve analysis), for which the present sample size was too small.

## Conclusions

To the best of our knowledge, the current study was the first quantitative, intensive longitudinal study applying an ambulatory assessment design investigating the transition period of patients after psychiatric hospitalization to school, focusing on patient–parent–teacher triads. As successful transition to school is assumed to be crucial for post-discharge adjustment [8], we aimed to gain a better understanding by investigating patients’ well-being on school days. We further pursued to identify variables across diagnosis being beneficial for patients’ well-being, allowing interventions to address those, and hence alleviate problems during the reintegration period.

The present results show that patients exhibited a decline in well-being and self-control during the weeks following discharge. Further, patients’ well-being, self-control, and academic self-efficacy were subject to considerable fluctuation over time. Meaningful differences between patients on the within-person level underpin the importance of ambulatory assessment studies and multilevel modeling and advocate an individualized analysis of needs and strengths of patients and their environments. Flexible interventions tailored to individual needs are indicated, as a general solution fitting all may not exist [10]. On average, to positively influence the transition process and patient’s well-being, it seems beneficial to support patients’ self-control and academic self-efficacy, as well as self-efficacy of relevant attachment figures such as parents. The present study indicates with a multi-perspective view how complex and challenging the transitional period after a psychiatric hospitalization is and how important it is to offer accompaniment and support to all parties involved during this period.

## Supplementary Information

**Additional file 1.** Daily Measures.

**Additional file 2.** Mixed models equations.

**Additional file 3.** Multilevel analyses.

## Data Availability

The datasets generated and analyzed during the current study are not publicly available due to the confidentiality of patient-related health data but are available from the corresponding author on reasonable request.

## Abbreviations

Kid-KINDL^R^: German questionnaire for measuring health-related quality of life in children
SCS-K-D: Brief German version of the Self-Control Scale
WIRKSCHUL: German scale school-related self-efficacy
*M*: Mean
*SD*: Standard deviation
*SE*: Standard error
α: Cronbach’s alpha
ω: McDonald’s omega
AR(1): First-order autoregressive error structure
ICC: Intraclass correlations
ISD: Intra-individual standard deviation
MSSD: Mean square successive difference
SC: Self-control
SE: Academic self-efficacy
SE_p_: Parental self-efficacy
SE_t_: Teacher self-efficacy
