# Supplementary material for: Patients’ well-being during the transition period after psychiatric hospitalization to school: insights from an intensive longitudinal assessment of patient–parent–teacher triads
Source: BMC Psychol. 2023 Jun 16;11:182. doi: 10.1186/s40359-023-01197-0 (PMC10276385; doi:10.1186/s40359-023-01197-0)
Supplement: Supplementary file 1 — Additional file 1. Daily Measures. [file 40359_2023_1197_MOESM1_ESM.docx]

## **Additional file 1 – Daily Measures**

Well-being

How were you overall today?

Self-control

Today, I have done something, I regretted later.(-)

I was lazy today.(-)

Today, I was able pulling myself together.

I had trouble concentrating today.(-)

Academic self-efficacy

Today, I was able to solve even complex tasks in class, when I made an effort.

Today, it was easy for me to comprehend things in class.

Today, at school I thought „ I can do it“.

Negative event

Did something happened today that you thought was bad at school?

If something like that happened today, what was it?

Positive event

Did something happened today that you thought was good at school?

If something like that happened today, what was it?

Parental self-efficacy

Today, I was able to guide my child also in problematic situations.

Today, I was able to come into good contact with my child, when I made effort to do so.

Today, I could not arrange much for the development of my child although I engaged much.(-)

Teacher self-efficacy

Today, I was convinced that I can teach the child the subject material also in problematic situations.

Today, I was convinced that I can come into good contact with the child, when I make effort to do so.

Today, I knew that I can not arrange much, although I engage in the development of the child.(-)
