# Supplementary material for: Patients’ well-being during the transition period after psychiatric hospitalization to school: insights from an intensive longitudinal assessment of patient–parent–teacher triads
Source: BMC Psychol. 2023 Jun 16;11:182. doi: 10.1186/s40359-023-01197-0 (PMC10276385; doi:10.1186/s40359-023-01197-0)
Supplement: Supplementary file 2 — Additional file 2. Mixed models equations. [file 40359_2023_1197_MOESM2_ESM.docx]

## **Additional file 2 – Mixed models equations**

Model 1:

Level 1 well-being_ij_ = β_0i_ + β_1i_ (negative event_ij_) + β_2i_ (positive event_ij_) + β_3i_ (day_ij_) + β_4i_ (within self-control_ij_) + β_5i_ (within academic self-efficacy_ij_) + *r*_ij_

Level 2 β_0i_ = γ_00_ + γ_01_(between self-control_i_) + γ_02_(between academic self-efficacy_i_) + μ_0i_

β_1i_ = γ_10_

β_2i_ = γ_20_

β_3i_ = γ_30_ + μ_3i_

β_4i_ = γ_40_ + μ_4i_

β_5i_ = γ_50_ + μ_5i_

Model 2:

Level 1 self-control_ij_ = β_0i_ + β_1i_ (day_ij_) + *r*_ij_

Level 2 β_0i_ = γ_00_ + μ_0i_

β_1i_ = γ_10_ + μ_1i_

Model 3:

Level 1 academic self-efficacy_ij_ = β_0i_ + β_1i_ (day_ij_) + *r*_ij_

Level 2 β_0i_ = γ_00_ + μ_0i_

β_1i_ = γ_10_ + μ_1i_

Model 4:

Level 1 well-being_ij_ = β_0i_ + β_1i_ (negative event_ij_) + β_2i_ (positive event_ij_) + β_3i_ (day_ij_) + β_4i_ (within parental self-efficacy_ij_) + *r*_ij_

Level 2 β_0i_ = γ_00_ + γ_01_(between parental self-efficacy_i_) + μ_0i_

β_1i_ = γ_10_

β_2i_ = γ_20_

β_3i_ = γ_30_ + μ_3i_

β_4i_ = γ_40_ + μ_4i_

Model 5:

Level 1 well-being_ij_ = β_0i_ + β_1i_ (negative event_ij_) + β_2i_ (positive event_ij_) + β_3i_ (day_ij_) + β_4i_ (within teacher self-efficacy_ij_) + *r*_ij_

Level 2 β_0i_ = γ_00_ + γ_01_(between teacher self-efficacy_i_) + μ_0i_

β_1i_ = γ_10_

β_2i_ = γ_20_

β_3i_ = γ_30_ + μ_3i_

β_4i_ = γ_40_ + μ_4i_
