# Supplementary material for: Patients’ well-being during the transition period after psychiatric hospitalization to school: insights from an intensive longitudinal assessment of patient–parent–teacher triads
Source: BMC Psychol. 2023 Jun 16;11:182. doi: 10.1186/s40359-023-01197-0 (PMC10276385; doi:10.1186/s40359-023-01197-0)
Supplement: Supplementary file 3 — Additional file 3. Multilevel analyses. [file 40359_2023_1197_MOESM3_ESM.docx]

## **Additional file 3 – Multilevel analyses**

| **Well-being (m1)** |  |  |  |  |  |  |  |  |  | Final model |  |
| --- | --- | --- | --- | --- | --- | --- | --- | --- | --- | --- | --- |
| *Fixed effects* |  |  |  |  |  |  |  |  |  |  |  |
| Intercept | 4.249 (0.107) | 4.336 (0.100) | 4.220 (0.106) | 4.360 (0.121) | 4.353 (0.118) | 4.317 (0.115) | 4.357 (0.120) | 4.413 (0.121) | 4.396 (0.117) | 4.397 (0.116) | 4.398  (0.111) |
| Negative event |  | -0.569* (0.082) | -0.576* (0.082) | -0.579* (0.082) | -0.592* (0.081) | -0.531* (0.081) | -0.515* (0.080) | -0.483* (0.079) | -0.482* (0.077) | -0.474* (0.077) | -0.472* (0.077) |
| Positive event |  |  | 0.254* (0.070) | 0.232* (0.071) | 0.242* (0.070) | 0.215* (0.070) | 0.198* (0.068) | 0.148* (0.067) | 0.144* (0.066) | 0.149* (0.065) | 0.139* (0.066) |
| Day |  |  |  | -0.006* (0.003) | -0.006* (0.004) | -0.004* (0.004) | -0.005* (0.004) | -0.007* (0.004) | -0.006* (0.004) | -0.007* (0.004) | -0.007* (0.004) |
| SC_within_ |  |  |  |  |  | 0.235* (0.046) | 0.218* (0.066) | 0.157* (0.068) | 0.168* (0.067) | 0.178* (0.064) | 0.178* (0.066) |
| SE_within_ |  |  |  |  |  |  |  | 0.244* (0.035) | 0.201* (0.058) | 0.207* (0.057) | 0.207* (0.057) |
| SC_between_ |  |  |  |  |  |  |  |  |  | 0.602* (0.165) | 0.459* (0.190) |
| SE_between_ |  |  |  |  |  |  |  |  |  |  | 0.119 (0.108) |
| *Random effects* |  |  |  |  |  |  |  |  |  |  |  |
| Intercept | 0.496 | 0.459 | 0.464 | 0.463 | 0.466 | 0.450 | 0.485 | 0.495 | 0.479 | 0.476 | 0.441 |
| Day |  |  |  |  | 0.016* | 0.016* | 0.017* | 0.016* | 0.016* | 0.016* | 0.016* |
| SC_within_ |  |  |  |  |  |  | 0.224* | 0.237* | 0.226* | 0.211* | 0.215* |
| SE_within_ |  |  |  |  |  |  |  |  | 0.212* | 0.206* | 0.206* |
| Residual | 0.869 | 0.845 | 0.835 | 0.831 | 0.792 | 0.780 | 0.767 | 0.743 | 0.723 | 0.724 | 0.724 |
| *logLik* | -1112.071 | -1088.764 | -1082.294 | -1079.575 | -1065.530 | -1052.843 | -1044.452 | -1020.633 | -1010.256 | -1005.494 | -1005.154 |
| AR(1) | 0.281 | 0.272 | 0.257 | 0.251 | 0.164 | 0.163 | 0.152 | 0.144 | 0.123 | 0.122 | 0.125 |

| **Self-control (m2)** |  |  | Final model |
| --- | --- | --- | --- |
| *Fixed effects* |  |  |  |
| Intercept | 4.031 (0.080) | 4.196 (0.091) | 4.198 (0.101) |
| Day |  | -0.008* (0.002) | -0.008* (0.004) |
| *Random effects* |  |  |  |
| Intercept | 0.374 | 0.374 | 0.459 |
| Day |  |  | 0.018* |
| Residual | 0.645 | 0.636 | 0.579 |
| *logLik* | -856.179 | -848.934 | -817.826 |
| AR(1) | 0.308 | 0.286 | 0.137 |

| **Academic Self-efficacy (m3)** |  |  | Final model |
| --- | --- | --- | --- |
| *Fixed effects* |  |  |  |
| Intercept | 3.756 | 3.689 (0.152) | 3.674 (0.178) |
| Day |  | 0.003 (0.002) | 0.004 (0.004) |
| *Random effects* |  |  |  |
| Intercept | 0.687 | 0.686 | 0.833 |
| Day |  |  | 0.014* |
| Residual | 0.810 | 0.809 | 0.785 |
| *logLik* | -1072.906 | -1072.145 | -1066.239 |
| AR(1) | 0.277 | 0.275 | 0.231 |

| **Well-being (m4)** |  |  |  |  |  | Final model |  |  |
| --- | --- | --- | --- | --- | --- | --- | --- | --- |
| *Fixed effects* |  |  |  |  |  |  |  |  |
| Intercept | 4.264 (0.109) | 4.363 (0.101) | 4.259 (0.108) | 4.389 (0.123) | 4.377 (0.119) | 4.384 (0.119) | 4.383 (0.118) | 4.384 (0.120) |
| Negative event |  | -0.674* (0.093) | -0.683* (0.093) | -0.683* (0.093) | -0.686* (0.092) | -0.679* (0.092) | -0.658* (0.092) | -0.679* (0.092) |
| Positive event |  |  | 0.216* (0.080) | 0.120* (0.080) | 0.217* (0.079) | 0.209* (0.079) | 0.206* (0.079) | 0.209* (0.079) |
| Day |  |  |  | -0.006* (0.003) | -0.006* (0.004) | -0.006* (0.004) | -0.006* (0.004) | -0.006* (0.004) |
| SE_p,within_ |  |  |  |  |  | 0.138* (0.051) | 0.133* (0.068) | 0.138* (0.051) |
| SE_p.between_ |  |  |  |  |  |  |  | 0.063 (0.145) |
| *Random effects* |  |  |  |  |  |  |  |  |
| Intercept | 0.488 | 0.446 | 0.447 | 0.445 | 0.429 | 0.428 | 0.424 | 0.436 |
| Day |  |  |  |  | 0.017* | 0.017* | 0.017* | 0.017* |
| SE_p,within_ |  |  |  |  |  |  | 0.192 |  |
| Residual | 0.890 | 0.862 | 0.855 | 0.852 | 0.813 | 0.809 | 0.801 | 0.808 |
| *logLik* | -986.655 | -961.238 | -957.568 | -955.524 | -943.974 | -940.384 | -938.474 | -940.305 |
| AR(1) | 0.267 | 0.265 | 0.255 | 0.250 | 0.168 | 0.162 | 0.160 | 0.162 |

| **Well-being (m5)** |  |  |  |  | Final model |  |  |  |
| --- | --- | --- | --- | --- | --- | --- | --- | --- |
| *Fixed effects* |  |  |  |  |  |  |  |  |
| Intercept | 4.340 (0.127) | 4.438 (0.119) | 4.226 (0.130) | 4.430 (0.158) | 4.427 (0.140) | 4.430 (0.140) | 4.427 (0.140) | 4.404 (0.134) |
| Negative event |  | -0.603* (0.140) | -0.643* (0.134) | -0.632* (0.136) | -0.611* (0.135) | -0.593* (0.135) | -0.611* (0.135) | -0.583* (0.136) |
| Positive event |  |  | 0.461* (0.116) | 0.440* (0.116) | 0.409* (0.113) | 0.391* (0.113) | 0.409* (0.113) | 0.419* (0.112) |
| Day |  |  |  | -0.009* (0.004) | -0.009* (0.006) | -0.009* (0.005) | -0.009* (0.005) | -0.008* (0.005) |
| SE_t,within_ |  |  |  |  |  | 0.146 (0.096) |  |  |
| SE_t,between_ |  |  |  |  |  |  |  | 0.376 (0.215) |
| *Random effects* |  |  |  |  |  |  |  |  |
| Intercept | 0.481 | 0.437 | 0.441 | 0.448 | 0.358 | 0.359 | 0.358 | 0.301 |
| Day |  |  |  |  | 0.017* | 0.016* | 0.016* | 0.015* |
| SE_t,within_ |  |  |  |  |  |  | 0.001 |  |
| Residual | 0.858 | 0.832 | 0.804 | 0.792 | 0.755 | 0.751 | 0.755 | 0.758 |
| *logLik* | -394.903 | -385.929 | -378.273 | -375.595 | -369.099 | -367.938 | -369.099 | -367.738 |
| AR(1) | 0.320 | 0.294 | 0.253 | 0.226 | 0.156 | 0.152 | 0.156 | 0.164 |
